# Indicators Predicting Inpatient Mortality in Post-Stroke Patients Admitted to a Chronic Care Hospital: A Retrospective Pilot Study

**DOI:** 10.3390/healthcare10061038

**Published:** 2022-06-02

**Authors:** Masatoshi Koumo, Akio Goda, Yoshinori Maki, Kouta Yokoyama, Tetsuya Yamamoto, Tsumugi Hosokawa, Junichi Katsura, Ken Yanagibashi

**Affiliations:** 1 Department of Rehabilitation, Hikari Hospital, Shiga 520-0002, Japan; motor.6e@gmail.com (M.K.); passatempo19840816@gmail.com (Y.M.); kotq_llo5@yahoo.co.jp (K.Y.); omusubikorori32@gmail.com (T.Y.); e_hunt_kun@yahoo.co.jp (T.H.); junichik1967@gmail.com (J.K.); kenyanagibashi@msn.com (K.Y.); 2 Department of Physical Therapy, Faculty of Health Sciences, Kyoto Tachibana University, Kyoto 607-8175, Japan; 3 Department of Neurosurgery, Hikone Chuo Hospital, Shiga 522-0054, Japan

**Keywords:** post-stroke, chronic phase, Barthel index, mortality, older adult, male human

## Abstract

Evidence concerning the mortality of post-stroke patients admitted to a chronic-phase hospital seems to be lacking. This pilot study aimed to identify mortality-related clinical variables in the admission of post-stroke patients from a retrospective perspective. A group of 38 non-survival stroke patients and another group of 46 survival stroke patients in a chronic-phase ward of the single center were recruited. Clinical variables including age, sex, stroke type, and Barthel index (BI) score were collected. The difference in the age and BI scores on admission were statistically significant between the two groups (*p* < 0.01). Polytomous logistic regression analysis revealed that age (odds ratio = 1.09, *p* = 0.03, and 95% confidence interval: 1.01–1.07), male sex (odds ratio = 5.04, *p* = 0.01, and 95% confidence interval: 1.39–18.27), and BI scores on admission (odds ratio = 0.90, *p* = 0.01, and 95% confidence interval: 0.83–0.97) could be prognostic variables. The percentage of correct classification was 83.3%. Age, male sex, and BI scores on admission may be prognostic indicators. The result of this study could lay the groundwork for palliative care for such a clinical population.

## 1. Introduction

Stroke is a major cause of bedridden status among patients in the aging Japanese society [1,2,3,4]. This may be because older stroke patients have more disabling strokes [5], leading to dependency, handicap, and long-term functional decline [6]. Japan has a large number of patients with stroke—more than one million as of 2017 [7]— which has had a considerable impact on the medical care industry. 

After post-stroke, patients undergo rehabilitation therapy at an acute phase hospital, the patients can be directly discharged to their home, transferred to a recovery phase hospital, chronic phase hospital, or a facility for senior citizens [8]. The post-stroke patients, who require substantial support for activities of daily living (ADL) due to impaired physical and cognitive performance, tend to be transferred to a chronic care hospital [9,10].

In Japan, stroke is the third leading cause of death after cancer and heart disease [11]. Therefore, mortality after stroke is a concern for clinicians, and research on this topic is ongoing [12,13,14,15]. In a 2-year study by Takashima et al. [12] involving Japanese patients with first-ever stroke, older age, male sex, history of stroke, higher Japan Coma Scale scores on admission, and subtypes of stroke (such as cerebral infarction, intracerebral hemorrhage, and subarachnoid hemorrhage) were associated with a risk of all-cause mortality. Moreover, Mutai et al. [14] reported that coronary artery disease and the motor independence index were associated significantly with mortality in post-stroke patients discharged home from a convalescent rehabilitation ward. Thus, factors that predict mortality in patients with post-stroke sequelae have been reported at various stages of the disease. However, to the best of our knowledge, the clinical factors related to the mortality of post-stroke patients during admission into a chronic phase hospital have not been described.

The patients admitted to chronic care hospitals often die in the hospital during their stay. The prediction of death of such patients is important to maintain their dignity and to provide psychological care for their families and others involved [16]. Therefore, this study aimed to examine clinical factors and socio-demographic factors that could predict mortality in such cases. 

## 2. Materials and Methods

### 2.1. Participants

We collected the clinical information of 84 post-stroke patients hospitalized in our chronic phase ward from October 2013 to March 2020.

The ethical committee of Hikari Hospital approved this retrospective cohort study. The requirement for informed consent from patients and their families was waived by the Ethics Committee due to the study’s retrospective nature. Data was obtained from the patients’ medical records.

### 2.2. Collection of Clinical Data

The following variables of the patients were collected: age, sex, underlying stroke etiology (infarction/hemorrhage), location of stroke lesion (supratentorial/infratentorial), laterality of stroke lesion (right/left), nutrient intake on admission (oral/non-oral), and Barthel index (BI) score on admission. Patients with subarachnoid hemorrhage and those whose data could not be fully obtained were excluded from this study. The patients were then categorized into two groups: the survival group (those who survived while admitted) and the non-survival group (those who died while admitted). The time of admission (from stroke onset to admission to our hospital) for the overall study patients was 172.6 ± 458.3 (mean ± standard deviation) days. The total follow-up period (from admission to discharge or death) for the overall study patients was 291.2 ± 286.5 (mean ± standard deviation) days. The period from admission to death in the non-survivor group was 318.9 ± 340.1 (mean ± standard deviation) days. The clinical variables between the two groups were statistically analyzed.

### 2.3. Barthel Index (BI)

The patients’ ADL was evaluated with BI [17]. BI includes ten variables: feeding, bathing, grooming, dressing, bowel control, bladder control, toilet use, transfer, mobility, and stair use; these variables are further divided. For each variable, scores of 0, 5, 10, or 15 are allocated based on the patient’s ADL. The minimum and maximum BI scores are 0 and 100, respectively.

### 2.4. Statistical Analyses

The normal distribution of the collected data was confirmed with the Shapiro–Wilk test. The variables were compared between both groups using the chi-square test and Mann–Whitney test. Thereafter, polytomous logistic regression analysis (simultaneous method) was conducted to identify the clinical factors related to the death of post-stroke patients during admission in a chronic phase hospital. The dependent variable was survival/non-survival during admission, and the independent variables were the patients’ collected variables.

We utilized SPSS Version 24 (SPSS IBM Corp.; Armonk, NY, USA). The statistical significance level was defined as *p* < 0.05.

## 3. Results

Thirty-eight patients (men: women = 20:18) were enrolled into the non-survival group, while forty-six patients (men: female = 16:30) were enrolled into the survival group. The mean age ± standard deviation of the non-survival group and that of the survival group were 85.7 ± 10.8 (mean age ± standard deviation) and 82.0 ± 8.0 years old, respectively; the difference in age between the two groups was statistically significant (*p* = 0.01). There was no significant difference in sex, underlying stroke disease, location of stroke lesion, laterality of stroke lesion, and nutrient intake on admission between the two groups. The BI scores on admission were 3.3 ± 5.7 in the non-survival group and 18.8 ± 21.3 in the survival group. The difference in BI scores between the two groups was statistically significant (*p* < 0.01) (Table 1).

The polytomous logistic regression analysis also showed that age (odds ratio = 1.09, *p* = 0.03, and 95% confidence interval: 1.01–1.07) and BI scores on admission (odds ratio = 0.90, *p* = 0.01, and 95% confidence interval: 0.83–0.97) could be prognostic indicators for death during admission. Male sex was also a significant variable for death during admission, when compared to female sex (odds ratio = 5.04, *p* = 0.01, and 95% confidence interval: 1.39–18.27). The percentage of correct classification was 83.3% (Table 2).

## 4. Discussion

In this study, we identified clinical variables that could be related to the mortality of post-stroke patients hospitalized at a chronic phase hospital. Age, being a man, and low BI scores were identified as potential prognostic indicators for death during admission. To the best of our knowledge, there is no similar study focused on post-stroke patients admitted to a chronic care hospital.

Age was a prognostic indicator for mortality during admission in our study. Takashima et al. analyzed clinical factors that increase the 2-year and 5-year mortality after stroke in the Japanese population of 1.4 million persons [12,13]. In their study, patients with subarachnoid hemorrhage were also enrolled. In our study, patients with subarachnoid hemorrhage were not included because we wanted to clearly define the laterality of stroke: right side or left. Thus, the included etiology of stroke in our study was not completely consistent with that in the study of Takashima et al. According to their study on the mortality risk of post-stroke patients two years after stroke, those aged from 75 to 84 years and those aged 85 years old or more had a hazard ratio of 7.20 and 13.16, respectively, when compared to those aged less than 45 years old. Likewise, regarding mortality risk, five years after stroke onset, the post hemorrhagic stroke patients aged from 75 to 84 years and those aged 85 years old or more had a hazard ratio of 29.18 and 57.69, respectively, when compared to those aged less than 45 years old. The post-ischemic stroke patients aged 75 to 84 and those aged 85 years old or older had a hazard ratio of 15.67 and 37.03, respectively, when compared to those aged less than 45 years old [12,13]. In our study, the mean age of the patients in the non-survival group was 85.7 years old. The mean age of the patients in the survival group was 82.0 years, a relatively old age. Our study also suggests that more aged post-stroke patients may have a high risk of mortality in a chronic phase hospital.

With polytomous logistic regression analysis in our study, being male was identified as a predictor of mortality of post-stroke patients while hospitalized in a chronic-phase hospital. An association between male sex and high risk of 5-year mortality after an ischemic and hemorrhagic stroke was observed [13]. The male patients had a shorter life expectancy than the female patients [18]; this study finding and those from previous studies are consistent with life expectancy data. Several studies [19,20] have shown that mortality after stroke is higher in women than in men. The discrepancy with the results of this study may be due to differences in lifestyle and race [21] or limitations of the present study.

The BI score is a well-utilized battery to evaluate the patient’s ADL [22,23]. Hankey et al. reported that a 30-day survivor of stroke whose pre-stroke ADL was dependent (defined as ADL with a BI score of less than 20) had a high mortality over the next five years [24]. Walker et al. analyzed the correlation between impaired ADL expressed as BI score and mortality for a long follow-up period. In their study, severely impaired ADL (defined as BI scores of 0–14), evaluated immediately after stroke onset, can result in high mortality for a long period [25]. In our study, the patients were evaluated with BI scores while hospitalized. Thus, the evaluation timing of patients’ ADL was different from those of the preceding studies. In addition, the period from the stroke onset to admission to our hospital was not obtained either in our study. However, compared to the BI scores of the patients in the survival group, patients in the non-survival group had a significantly low mean BI score on admission (3.3). This suggests that patients with severely impaired, dependent ADL on admission have a high mortality risk during admission in a chronic phase hospital.

### Limitations

First, this study was a single-center retrospective study with a small sample size. Second, regarding subjective bias, multiple rehabilitation therapists evaluated the patients’ statuses with BI scores. Third, the cause of death during admission was not analyzed in this study. Fourth, the period from stroke onset to death was not examined in this study. Fifth, factors such as comorbidities, risk factors for stroke onset, lesion size, acute National Institutes of Health Stroke Scale score, time since stroke onset, and ADL before admission were not examined in this study. Owing to these limitations, the generalizability of our findings is limited to the source population. Despite these limitations, we believe that our findings offer valuable information to professionals in the field of chronic medical care and will be useful in providing family members and others involved with chronic post-stroke patients with the opportunity to visit them prior to their death.

## 5. Conclusions

In a post-stroke chronic phase hospital, aged male patients with low BI scores on admission could have high mortality. Clinicians should be aware of the possible high mortality of those patients, to take care of not only their patients, but their families as well.

## Figures and Tables

**Table 1:** The results of clinical and sociodemographic variables compared between the non-survival group and survival group.

	Non-Survival Group (*n* = 38)	Survival Group (*n* = 46)	*p* Value
**Age ☨** **(years)**	85.7 ± 10.8	82.0 ± 8.0	0.01
**Sex** **(male/female)**	(20/18)	(16/30)	0.12
**Nutrient intake on admission** **(oral/non-oral)**	(23/15)	(30/16)	0.82
**Underlying stroke etiology** **(Infarction/Hemorrhage)**	(36/2)	(42/4)	0.68
**Location of stroke lesion (Supratentorial/Infratentorial)**	(18/20)	(20/26)	0.83
**Laterality of stroke lesion (right/left)**	(16/22)	(29/17)	0.08
**BI score on admission** **☨**	3.3 ± 5.7	18.8 ± 21.3	<0.01

Mean ± standard deviation. Chi-square test, ☨: Mann–Whitney Test.

**Table 2:** Results of the polytomous logistic regression analysis.

	*p* Value	OR	95% CI
Age	0.03	1.09	1.01–1.17
Sex			
Male		5.04	
Female	0.01	1.00	1.39–18.27
Underlying stroke etiology			
Infarction		0.71	
Hemorrhage	0.58	1.00	0.21–2.37
Location of stroke lesion			
Supratentorial		0.85	
Infratentorial	0.90	1.00	0.07–10.32
Laterality			
Right		0.93	
Left	0.90	1.00	1.002–1.028
Nutrient intake on admission			
Oral		2.25	
Non-oral	0.22	1.00	0.61–8.27
Barthel index score on admission	0.01	0.90	0.83–0.97

OR: Odds ratio, 95% CI: 95% confidence interval.
